# Acute Post-streptococcal Glomerulonephritis in a Pediatric Population: A Five-Year Retrospective Study

**DOI:** 10.7759/cureus.56082

**Published:** 2024-03-13

**Authors:** Prayes Bajracharya, Ashish Khadgi, Sugandha Shrestha, Ramji Silwal, Anisha Tandukar

**Affiliations:** 1 Department of Pediatrics, Buddha International Hospital, Ghorahi, NPL; 2 Department of Ear, Nose, and Throat and Head and Neck Surgery, Lamahi Eye Hospital, Lamahi, NPL; 3 Department of Obstetrics and Gynaecology, Buddha International Hospital, Ghorahi, NPL; 4 Department of Pediatrics, Alka Hospital, Kathmandu, NPL; 5 Nursing, Rapti Academy of Health Sciences, Ghorahi, NPL

**Keywords:** gas, glomerulonephritis, psgn, outcome, post-streptococcal glomerulonephritis

## Abstract

Introduction: Post-streptococcal glomerulonephritis is a kidney disease that occurs after infection with a certain strain of streptococcal bacteria. It has a high hospitalization rate, especially in developing countries. It is characterized by the sudden appearance of edema, hematuria, proteinuria, and hypertension. The objective of this retrospective descriptive study conducted at James L. Gordon Memorial Hospital in Olongapo, Zambales, Philippines, is to analyze the demographic distribution, clinical presentation, complication, and outcome of acute post-streptococcal glomerulonephritis (APSGN) in a pediatric population.

Methods: A retrospective descriptive study was done in patients (below 18 years of age) admitted with the diagnosis of post-streptococcal glomerulonephritis during a five-year period in the pediatric ward of a tertiary hospital in the Philippines. All patients underwent detailed clinical history and examination and were closely monitored during treatment. The course of disease progression was closely monitored and recorded.

Results: Seventy-seven children were treated for APSGN with a mean age of 7.8 years and with standard deviation of 3.85. Edema and gross hematuria were noted in 53 (68.8%) and 24 (31.1%) cases, respectively. Hypertension was present in 54 patients (70.1%). Among those 54 patients, 49 (63.6%) patients developed acute renal injury (based on normal creatinine for age), three cases (3.8%) had pleural effusion, and two patients (2.5%) developed hypertensive encephalopathy. All patients underwent KUB (kidneys, ureters, and bladder) ultrasound, among which 13 (16.88%) had diffuse parenchymal swelling which improved and eventually were discharged. Increased blood urea nitrogen (BUN) was noted in 60-65% of patients. Hypoalbuminemia and lower hemoglobin were also quite common. There was no mortality during treatment and hospital stay.

Conclusions: APSGN remains one of the most common causes of glomerulonephritis in the region. Edema, hypertension, and hematuria were the most common presenting features. Early identification and comprehensive monitoring and management are required to prevent morbidity and mortality. The prognosis is generally good if early treatment is done.

## Introduction

Acute post-streptococcal glomerulonephritis (APSGN) is an immune-mediated disease associated with acute respiratory tract infection and skin infection caused by group A beta-hemolytic *Streptococcus*. Group A beta-hemolytic bacteria usually present with edema, hematuria, hypertension, and azotemia [1]. There is male predominance (2:1), and it most commonly affects children aged 3-10 years, while it is relatively uncommon before age three years [2]. The typical patient develops APSGN within 1-2 weeks after the antecedent streptococcal pharyngitis or 3-6 weeks after the streptococcal pyoderma [3,4]. Most cases of APSGN occur following pharyngitis with streptococci rather than skin infection [5]. Most children with APSGN recover, but some patients may develop complications including renal impairment, congestive heart failure, pulmonary edema, and encephalopathy [6].

APSGN clinical case is defined as having at least two of the following: facial edema, hypertension, moderate hematuria on dipsticks (2+ red blood cells) and/or peripheral edema with laboratory evidence of hematuria on microscopy (RBC >10/ml), evidence of recent streptococcal infection, i.e., positive group A streptococcal culture from the skin or throat, or elevated anti-streptolysin O (ASO) titer or anti-DNAse B and low C3 complement level (below the normal value). Confirmed cases are defined as cases with either laboratory definitive evidence (positive renal biopsy) or both clinical evidence and laboratory evidence. Probable cases require only clinical evidence, while possible cases require only laboratory evidence only (according to the Northern Territory guidelines) [7].

This study is done to analyze the demographic distribution, clinical presentation, complication, and outcome of APSGN in a pediatric population presented at James L. Gordon Memorial Hospital in Olongapo, Zambales, Philippines.

## Materials and methods

A retrospective descriptive study was conducted to evaluate all children presented in the pediatric ward of James L. Gordon Memorial Hospital in Olongapo, Zambales, Philippines, with the diagnosis of APSGN between January 1, 2013, and December 31, 2017. This study was conducted in accordance with the ethical standards of the responsible committee on human experimentation of the institution after ethical approval was granted by the Institutional Review Committee of James L. Gordon Memorial Hospital (approval number: 122). Consent was taken during the time of admission from the guardians of all patients, and confidentiality was maintained. All the medical records in the form of manual charts were reviewed for patients admitted with a discharge diagnosis of "post-streptococcal glomerulonephritis" during the study period. Charts of 96 patients were retrieved with the abovementioned diagnosis. Only 77 charts were included in the study because, in 15 charts, the diagnosis could not be verified due to the absence of pertinent laboratory results (because of financial constraints and loss of follow-up), two were excluded due to lupus nephritis, one was excluded due to nephrotic-nephritic disease, and one was excluded due to Henoch-Schönlein purpura nephritis. The 77 patients that were included in the study were local patients. Patients were identified as confirmed or probable. Socio-demographic data, clinical-laboratory profile, and outcomes were obtained from medical records, and descriptive analysis was carried out. Children were included if they presented with at least two signs of an acute nephritic syndrome (hematuria, edema, and hypertension) associated with the evidence of activation of an alternative pathway complement system (low C3 serum level), serological and direct evidence of previous or current streptococcal infection, i.e., positive group A streptococcal culture from the skin or throat, or elevated ASO titer or anti-DNAse B. Clinical evidence of streptococcal infection was assumed on either history of recent sore throat or clinical examination showing impetigo or scarring indicating healed recent impetigo. C3 complement and ASO titer were measured using nephelometry, and anti-DNAse was measured by using latex-enhanced nephelometry. Children found to have causes of acute glomerulonephritis other than APSGN were excluded. Information collected from medical records included demographic data, age at presentation, anthropometry, blood pressure, complete blood count, renal function tests, urinalysis, ultrasound of the abdomen, need for hospitalization, and details of treatment. The potential risk factors seen contributing to the occurrence of APSGN are socioeconomic standards and availability of access to clean water and sanitation. Hypertension in this study was defined as blood pressure >95th percentile for age, sex, and height. Hematuria in microscopy is defined as RBC >10/UL. Edema is defined as puffiness of the face, bilateral pitting pedal edema, and/or abdominal wall edema. Acute kidney injury (AKI) is defined as a clinical syndrome in which there is a sudden deterioration in renal function which results in the inability of the kidneys to maintain fluid and electrolyte homeostasis. Supportive therapy including salt and fluid restriction with loop diuretics was the first line of treatment. Anti-hypertensive medicines were used when deemed necessary.

Inclusion and exclusion criteria

Children below 18 years of age were included in the research who presented with symptoms of APSGN. On the other hand, those patients whose diagnosis could not be verified in the absence of pertinent laboratory results and those with other causes of glomerulonephritis (autoimmune disease, vasculitis, and other causes of infection besides group A *Streptococcus*) were excluded from the study.

Data was collected and analyzed using IBM SPSS Statistics for Windows, Version 25.0 (Released 2017; IBM Corp., Armonk, New York, United States).

## Results

**Figure 1:**
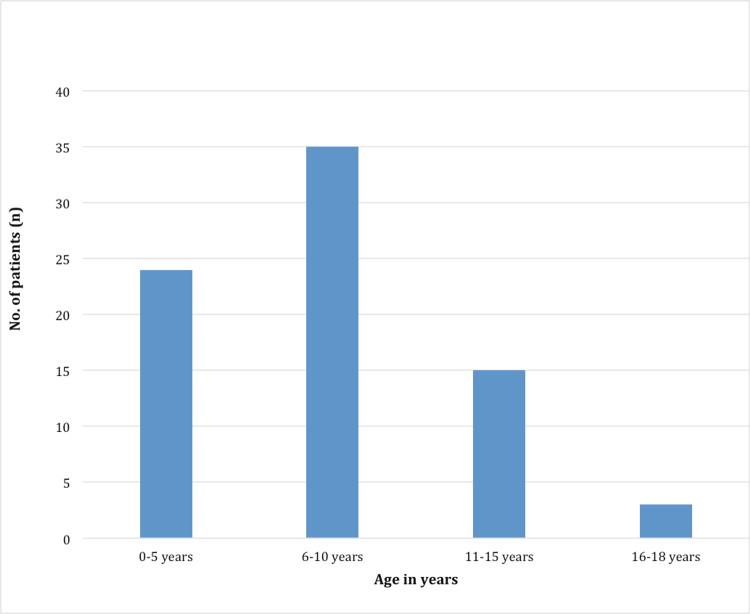
Distribution of patients according to age

In this study, a total of 77 patients were identified as APSGN in a tertiary hospital in Olongapo, Zambales, Philippines, during the study period. The ages of the patients ranged from 2 to 18 years (Figure 1). Twenty-four patients belonged to the age group five years and below, 35 belonged to the age group 6-10 years, 15 belonged to the 11-15 age group, and three belonged to the age group 16-18 years. We had two young patients, both were two years old, one was identified as a confirmed case, and another as a probable case of APSGN. Age group 6-10 years was the most affected age group, and the mean age of presentation was 7.8 years with a standard deviation (SD) of 3.85.

**Figure 2:**
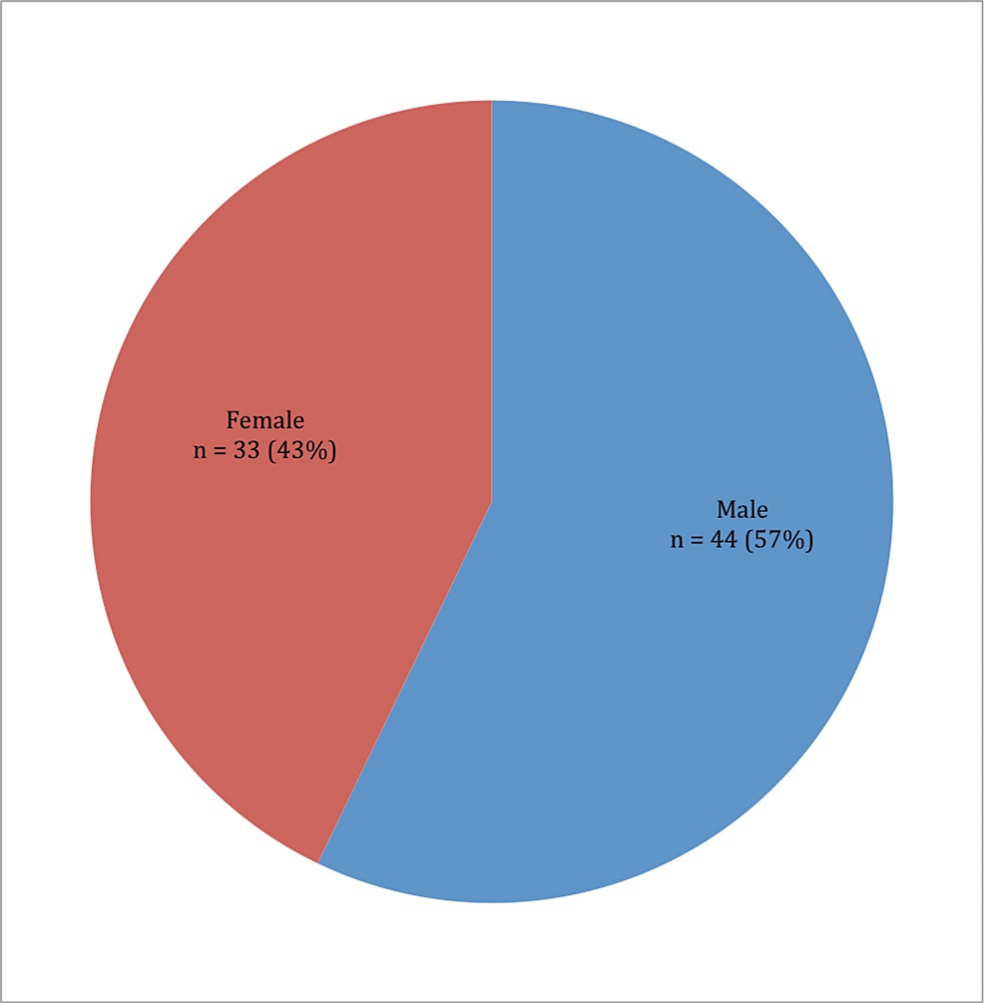
Distribution of patients according to sex

Among the patients, 44 (57%) were males, and 33 (43%) were females (Figure 2). There was male predominance, and the male-to-female ratio was 1.3:1.

The most common presentation in all age groups was hypertension and edema followed by gross hematuria except in the 16-18 age group in which hypertension and edema were the most common presentation followed by dizziness and seizure (Table 1). Edema and gross hematuria were noted in 53 (68.8%) and 24 (31.1%) patients, respectively. Hypertension was present in 54 (70.1%) patients, of which two of them (2.5%) developed hypertensive encephalopathy. Three patients (3.8%) patients developed dizziness with seizures.

**Table 1:** Most common presentation according to age group

Age group	No. of patients	Most common presentation
5 years and below	24	Hypertension and edema followed by hematuria
6-10 years	35	Hypertension and edema followed by hematuria
11-15 years	15	Hypertension and edema followed by hematuria
16-18 years	3	Hypertension and edema followed by dizziness and then seizure
	Total: 77	

Fifty-four patients developed some form of complications during treatment (Table 2). Forty-nine (63.6%) patients had acute renal injury (based on normal creatinine for age). Three cases (3.8%) had pleural effusion with spontaneous resolution and were discharged without further complication. Two patients (2.5%) developed hypertensive encephalopathy. Among them, in the eight-year-old female patient, who had four episodes of seizure, the cranial CT scan showed bilateral occipital subcortical ischemic changes, while in the 14-year-old male patient with one episode of seizure, the cranial CT scan showed acute infraction in both parietal lobes. These cases were referred to a neurologist which improved, and hence, they were discharged. One patient developed a seizure due to hyponatremia secondary to acute renal injury which was referred to a nephrologist and was discharged after improvement.

**Table 2:** Outcome of the patients

No. of patients	Complication	Outcome
49 (63.6%)	Acute renal injury	Referred to a nephrologist. Patients improved after treatment.
3 (3.8%)	Pleural effusion	Spontaneous resolution and discharged.
2 (2.5%)	Hypertensive encephalopathy	Referred to a neurologist for CT head changes. Patients improved and hence were discharged.
Total: 54		

All 77 patients underwent KUB (kidneys, ureters, and bladder) ultrasound; among them, 13 (16.88%) had diffuse parenchymal swelling, which improved after treatment. Nineteen (24.6%) out of 77 were identified as confirmed cases, while 58 (75.3%) were identified as probable cases. All confirmed cases of APSGN were associated with low serum C3 levels, hematuria, proteinuria, and increased ASO titer.

There was not any mortality during treatment and hospital stay.

## Discussion

Post-streptococcal glomerulonephritis remains an important non-suppurative complication of group A beta-hemolytic streptococcal infection worldwide. It is estimated that the worldwide prevalence of APSGN is 472,000 cases, out of which 404,000 of those cases occur in children [8]. The apparent decline in the incidence of APSGN in developed countries is due to the near eradication of streptococcal pyoderma due to better hygiene [9]. The prevalence of streptococcal infections is low in developed nations due to the disinfection of water with the use of fluorination, because of which the virulence factor of the bacteria was decreased. People also had good and early access to medical treatment [10].

APSGN rarely occurs before age two years. The rarity of APSGN in very young children was attributed to the low rates of streptococcal pharyngitis in this age group and immature immune (or antibody) response [11]. In our study, we identified two patients who were two years old. The male-to-female ratio was 1.3:1 which is similar to another study [12].

The most common age group was 3-10 years, which was comparable with another study [3]. The most common presenting feature in this age group was edema in 53 (68.8%) and gross hematuria in 24 (31.1%) patients, which was comparable to a study done by Maalej et al. in which edema and hematuria were noted in 135 (75.8%) and 114 (64%) cases, respectively. Forty-two (54.5%) developed acute renal injury (creatinine based on age), whereas in a Tunisia study, 37% developed renal injury [13].

Glomerular hematuria is almost a universal finding. Our study showed 31.1% with gross hematuria which is comparable to another study [13]. While dyspnea may be a presenting feature in around 5% of patients done in a study by Burke and Titus, there were no patients in our study with complaints of dyspnea [14]. Increased blood urea nitrogen (BUN) was noted in 60-65% of patients done in a study by Burke and Titus which was much closer to our study with a 57% increment in BUN. Hypoalbuminemia and lower hemoglobin were also quite common [14].

Treatment is usually supportive, and the most urgent problem like hypertension should be addressed early. Salt restriction and loop diuretics are the first-line treatment for fluid overload and hypertension. Some studies have shown that treating communities in which APSGN is epidemic with benzathine penicillin G may reduce the carriage of nephritogenic strains and thus lower the incidence of APSGN [15].

Renal biopsy is indicated in the case of a diagnostic dilemma to confirm or exclude the disease when there are atypical features (presence of acute renal failure, nephrotic syndrome), rapid progression or inadequate recovery, or where an alternative diagnosis has to be considered, while in our study renal biopsy was not done as patients improved with treatment. The recovery phase occurs after the resolution of the fluid overload with diuresis either pharmacologically or spontaneously. Proteinuria resolved before hematuria in most of our patients [16]. Recurrence is rare which was not seen in our research [17]. 

We are aware of the limitation of our study being retrospective with possible errors in data collection, missed cases, and potential bias of the cases recalled by clinicians. We were unable to collect environmental data for the chart otherwise that would portray the significance of causative factors such as overcrowding and sanitation in transmission and occurrence of the disease.

For future researchers and clinicians, we recommend throat swabs be taken in all patients suspected of APSGN. For the prevention of APSGN and its complications, community awareness programs should be done on a regular basis emphasizing the importance of personal hygiene and sanitation. People should also be educated to seek professional medical help for throat pain and skin infections.

## Conclusions

Though a declining trend of APSGN is evident, it is still a health problem in less developed and developing countries. Onset of edema and hematuria are still the most common presenting features of this disease. No significant difference was observed when this study was compared to local and foreign studies. The immediate outcome of the patient with APSGN is excellent; however, a sequential follow-up is essential to know the long-term consequences of the disease and to prevent further complications. Thus, we recommend routine throat swabs be taken in all patients suspected of APSGN and community awareness programs be done on a regular basis for the prevention of this disease.
